# Conference Report: WORKSHOP ON THIN FILM THERMAL CONDUCTIVITY MEASUREMENT AT THE THIRTEENTH SYMPOSIUM ON THERMOPHYSICAL PROPERTIES Boulder, CO June 25–26, 1997

**DOI:** 10.6028/jres.103.006

**Published:** 1998-02-01

**Authors:** Albert Feldman, Naira M. Balzaretti

**Affiliations:** National Institute of Standards and Technology, Gaithersburg, MD 20899-0001

## 1. Introduction and Conclusions

A Workshop was held on the subject of Thin Film Thermal Conductivity Measurement as part of the Thirteenth Symposium on Thermophysical Properties at the University of Colorado in Boulder CO, June 25–26, 1997. The purpose of this Workshop was to provide a forum for producers and users of thermal properties data to discuss the unique problems associated with measuring the thermal conductivities of thin films and coatings. The workshop was organized by Albert Feldman of the National Institute of Standards and Technology, David Cahill of the University of Illinois at Urbana-Champaign, and William P. Allen of the United Technologies Research Center.

The wide use of thin films and coatings inevitably places many of these layered structures in situations that require them either to conduct heat easily or to impede the flow of heat. In many instances, the thermal performance of a film will determine whether a part or device will fail if the film thermal conductivity does not fall within a specified range of values. Due to morphological differences, the thermal conductivity (*κ*) of a film can be significantly less than the thermal conductivity of a bulk material having the same nominal composition. In addition to having a modified thermal conductivity, the thermal conductivity of thin films may exhibit anisotropy and inhomogeneity. Furthermore, the effect of the interfacial thermal resistance (ITR) between the film and the substrate takes on greater importance as films become thinner. Techniques for measuring the thermal conductivities or thermal diffusivities of films and coatings and the ITR in layered structures become very important because the data generated make it possible for system engineers and designers to predict the performance of these structures in practical applications. It is also desirable to employ techniques that are easily and rapidly implemented, because the thermal conductivity of a film with a given nominal composition may not be reproducible due to morphological variations that occur during deposition, thus making it advisable to measure the thermal conductivity of each individual film.

Approximately 45 people attended most sessions of the Workshop. There were six sessions; four of the sessions were devoted to 17 oral presentations and two of the sessions were devoted to discussions of the two principal subjects of the Workshop. The principal subjects were: (1) methods and problems associated with measuring the thermal conductivity of thin films, and (2) measuring the thermal conductivity of chemical vapor deposited (CVD) diamond. Below are reviews of each of the presentations. Prior to each review we give the title of the presentation and the list of coauthors. In four instances, we present only the abstract submitted by the authors. For additional information regarding the presentations, the reader should contact the presenting authors.

A voluntary questionnaire was distributed to Workshop attenders. Twenty five responses were received. The questionnaire requested information regarding the relationship of the respondent to thermal conductivity measurement, the relevance of the Workshop to the respondent, and the willingness of the respondent to participate in a collaborative activity such as standards development. The results are given in the Appendix.

The purpose of this review is to describe some of the results presented at the Workshop and to draw some conclusions that might prove useful. The conclusions are summarized below:

1. Most attenders felt that thermal conductivity measurements on thin films require smaller uncertainty and greater reliability than reported. There was some controversy as to how much the thermal conductivity of a thin film differs from that of the bulk material of the same nominal composition and also the degree to which the thermal conductivity varies with film thickness. Since the reliability of a measurement frequently cannot be determined, many of the Workshop attenders agree to participate in round-robin measurement comparisons. A layer of silicon dioxide produced by oxidation of silicon was recommended as a good specimen for a round-robin and also possibly as a reference material for thin-film thermal conductivity. This would be a material having a low thermal conductivity; there was no consensus on the choice of a thin-film material of high thermal conductivity to be used as a reference material.
2. Many attenders disagreed on the meaning of an interface. Researchers should therefore define what they mean by an interface when describing their experiments. There is a need to characterize the region between a film and a substrate because unwanted phases may be present that adversely affect thermal conductivity measurements. Whether to call this region an interface is a matter of definition.
3. The measurement of thermal conductivity in CVD diamond is much less reliable than expected as determined by an interlaboratory round-robin comparison. Measurements made by the dc heated bar methods appeared to agree well with each other, but the ac measurements gave a large spread in values; uncertainties claimed by many of the participating laboratories are much smaller than the interlaboratory spread. The source of these discrepancies requires further investigation.

## 2. Thin Film Thermal Conductivity

*Thermal Conductivity of Thin Films Via the Thermal Comparator Method*, J. C. D. Lambropoulos, Department of Mechanical Engineering, University of Rochester, Rochester, NY 14627, USA.

J. C. D. Lambropoulos reviewed the importance of thermal conductivity measurements for predicting damage thresholds in films subjected to high-power laser radiation. Part of his discussion described the thermal comparator method for measuring thin-film thermal conductivity and ITR. The thermal comparator consists of a conical tip that is held in contact with the specimen surface. The thermal conductivity of the specimen is calculated from the temperature evolution resulting from a heat pulse applied to the tip. A particular limitation of the method is the difficulty in making the thermal contact resistance between the tip and the specimen surface reproducible. To study the ITR, a microstructural interface model was used, taking into account the interface, diffuse porosity, and the varying microstructure of the film near the interface. Experimental results for oxide and other ceramic films on several substrates indicated that the thermal conductivities of the films were significantly smaller than the thermal conductivities of the corresponding bulk materials. All of the films also showed a significant ITR. The ITR was attributed to morphological structure at the interface that contained a significant concentration of grain boundaries and voids. The columnar structures typically observed in cross-sectional images of films would account for these defects. From discussions during the question period, one can conclude that the thermal comparator method is not a desirable method of measuring the thermal conductivities of thin films and ITR.

*Importance of Thermophysical Properties of Thin Films in ULSICs and Beyond*, A. N. Saxena, International Science Company, 4217 Pomona Avenue, Palo Alto, CA 94306, USA.

A. N. Saxena reviewed the importance of thermal conductivity to the performance of ultra-large-scale integrated circuits (ULSICs). The thermal conductivities of the material components determine how rapidly heat can be removed from a device. If sufficient heat cannot be removed, excessive build-up of temperature can occur leading to failure of the device from electromigration and from thermally-induced stress. The problem is further exacerbated because the thermal conductivities of many of the materials are lower than expected because the materials are in the form of thin films which are known to have lower thermal conductivity values than those of the equivalent bulk materials. The paper considered the particular importance of silicon dioxide films which are used as interlayer dielectric films (ILDs). Saxena showed that the effective thermal conductivity of the silicon dioxide depended on the method of film growth and on the film thickness. In all cases, the thermal conductivity of the film was lower than the thermal conductivity of the bulk material; furthermore, the thermal conductivity decreased with decreasing film thickness, *d*; the thermal conductivity was found to depend on thickness as *κ*~*d*^−1/2^, according to the model of Savvides and Goldsmid [N. Savvides and H. J. Goldsmid, Phys. Lett., **41A**, 193 (1972)]. An extrapolation to infinite thickness gives the effective bulk value of the film thermal conducitivity, *κ*_e_. Thermal annealing appeared to densify the films, thus increasing *κ*_e_; thermally grown silicon dioxide had *κ*_e_ closest to the bulk value. Thus, *κ*_e_ becomes a measure of the quality of the film. The slope of the linear dependence described above appears to be correlated with the adhesion of the film; a smaller slope indicates a smaller contribution of the interface to thermal conductivity, suggesting better adhesion. The principal conclusion drawn from the paper is that the performance of a device can best be predicted if the thermal conductivity of the silicon dioxide ILD’s is known; the best performance will occur when the thermal conductivity of the silicon dioxide has the bulk value and when the ITR is small or negligible.

*Confirmation of the Thickness Effect Observed in the Transverse Thermal Conductivity of Thin Amorphous Films Deposited on Silicon*, L. P. Hehn, F. R. Brotzen, and D. L. Callahan, Department of Mechanical Engineering and Materials Science, Rice University, Houston, TX 77005-1892, USA and P.J. Loos, Texas Instruments Inc., Houston, TX 77251-1443, USA.

*Submitted abstract:* The measurement of transverse thin-film thermal conductivity is complicated by a lack of standardization. A silicon standard has been used to verify the temperatures and temperature distribution determined by thermocouple measurements [P. R. Brootzen, P. J. Loos, and D. P. Brady, Thin Solid Films **207**, 197–201 (1992)]. As a result of this standardization, geometrical improvements have been made in the thin-film transverse thermal conductivity measurement device. Verification of measurements in the Al/amorphous Si0_2_/Si substrate system proves that the large thermal conductivity drop observed with decreasing Si0_2_ thickness is a material effect as opposed to an artifact in measurement. The possibility of thickness-dependent thermal conductivity in metallic layers was examined. An attempt was made to produce a thermally resistant interfacial boundary through the addition of one and two thin layers of thin Ti:W and AlCu films within the thin-film dielectric structure. The metal layers were added to examine the thermal conduction mismatch with the dielectric layers (i.e., electrons dominate thermal energy transport in metals whereas phonons dominate in the dielectric layers). The metal layers were found to provide no added thermal boundary resistance and the thermal conductivity of the combined multi-layered system being similar to that with no metal layers.

*Measurement of Thermal Conductance of Microcrack Interfaces in Composite Materials*, K. McDonald, J. Dryden, F. Zok, Department of Materials, University of California, Santa Barbara, CA 93106, USA, A. Majumdar, Department of Mechanical Engineering, University of California, Berkeley, CA 94720, USA.

*Submitted abstract:* Application of stress on ceramic-matrix fiber composites can lead to formation of microcracks in the matrix and debonding between the fiber and the matrix. The additional interfaces created by such processes can reduce the effective thermal conductivity of these materials. In applications involving high heat fluxes and thermal stresses such as in combustor linings, reduction of thermal conductivity by microcracks can lead to higher temperature gradients, higher thermal stresses, more matrix cracking, and eventually catastrophic failure. For the purpose of design, therefore, it is important to quantify the reduction of thermal conductivity due to microcracks. This paper will present some of the first quantitative measurements of the conductance of microcracks as a function of crack opening displacement. The data will be used to understand the role of fibers in thermal bridging across the cracks.

*Heat Transport in Thin Films with Shape Memory*, E. Quandt and M. Rohde, Forschungszentrum Karlsruhe, Institut für Materialforschung I, P.O. box 3640, 76021 Karlsruhe, Germany.

*Submitted abstract:* Materials with shape memory as the NiTi alloys are gaining importance for applications in microsystems and thin-film technology. Shape memory alloys are used to fabricate microgrippers or microactuators. Since the shape memory effect is induced by heating or cooling the material over characteristic transition temperatures, the thermophysical properties like the thermal conductivity and the heat capacity determine the behavior of the temperature field in space and time and therefore its operational performance. The transition temperatures of these alloys can be adjusted from room temperature up to 500 °C by adding Pd or Cu and reducing the Ni content. Measurements of the thermal diffusivity and the heat capacity have been performed on thin films of NiTi, NiTiCu, NiTiPd, and TiPd as a function of temperature in order to evaluate the changes of these properties below and above the phase transition associated with the shape memory effect. The thickness of the free standing films which have been fabricated by a magnetron sputtering technique is in the range between 10 μm and 13 μm. The thermal diffusivity has been determined with a photothermal method within an experimental setup which allows for sample heating up to 500 °C. The heat capacity has been measured with a Perkin-Elmer DSC. From the experimental data of the density, the thermal diffusivity and the specific heat, the thermal conductivity values of the films could be calculated. The heat capacity of the films changes only slightly with temperature with exception of the transition region where it exhibits strongly pronounced peaks. Temperature hysteresis could be observed on heating and cooling of the sample. The thermal conductivity is significantly reduced in the NiTi film compared to a 500 μm thick NiTi foil. The behavior of the thermal conductivity as a function [of the] temperature in the film differs also from that of the foil. Up to the transition temperature its value decreases and starts to increase after passing the phase transition. Whereas in the foil an increasing thermal conductivity could be observed on heating with no changes in slope at the transition temperature. Changing the stoichiometry of the films by adding Cu or Pd and reducing the Ni content [affects] the transition temperature, [the] temperature hysteresis and [the] temperature dependent behavior of the thermal conductivity.… The experimental results will be discussed within a framework of a heat transport model for the shape memory alloys.

*Thermal Properties of Novel Thin Films in ICs and MEMS*, K. E. Goodson, M. Asheghi, M. N. Touzelbaev, K. Kurabayashi, Y. S. Ju, B. W. Chui, and T. W. Kenny, Department of Mechanical Engineering, Stanford University, Stanford, CA 94305-3030, USA.

K. E. Goodson described several high resolution techniques to measure the thermal properties of thin films used in integrated circuits and micro-electromechanical systems (MEMS). The thermal conductivity affects the performance and reliability of these devices. He employs scanning thermal microscopy using both far-field and near-field optics. Near-field optical thermometry is of particular interest because it overcomes the diffraction limit of conventional far-field techniques. Temperature can be measured with nanosecond temporal resolution and sub-wavelength spatial resolution. Measurements of the effective thermal conductivities of polymers and silicon dioxide have been made as a function of layer thickness. Most measurements give thin-film thermal conductivity values less than those of the bulk values. In the case of silicondioxide layers, thermal conductivity increases with increasing film thickness; after annealing, the thermal conductivity values of the thicker films are about equal to that of bulk silicon dioxide. The thermal conductivities of CVD diamond films were found to increase with thickness. In another experimental system that employed a three heater thermometer bridge, Goodson obtained the temperature dependence of the thermal conductivities of thin silicon layers that were part of thin silicon-on-insulator multilayers. Due to impurity-scattering and boundary-scattering the values of thermal conductivity were much less than that of bulk silicon.

*Scanning Thermal Microscopy of Novel Devices and Microstructures*, A. Majumdar, Department of Mechanical Engineering, University of California, Berkeley, CA 94720, USA, J. Varesi and Z. Shi, Department of Mechanical Engineering, University of California, Santa Barbara, CA 93106, USA, K. Luo, Anadigics Inc., 35 Technology Drive, Warren, NJ 07059, USA.

*Submitted abstract:* The scanning thermal microscope (SThM) uses a temperature-sensitive cantilever probe in an atomic force microscope (AFM) to simultaneously obtain topographical and thermal images of a material surface. The SThM has been used to map the temperature of individual Si and GaAs transistors as well as vertical-cavity surface emitting lasers. This paper will report the effects of device geometry, device operation condition as well as thermal and electrical properties, on the temperature rise and Joule heating in these devices. The mechanism of probe-sample heat transfer as well as the limits on spatial resolution will be discussed. A new spin-off from SThM, which can probe Joule heating below 50 nm spatial resolution, will also be illustrated.

*Heat Transport Measurements of Thin Films and Interfaces*, D. G. Cahill and S. M. Lee, University of Illinois, Urbana, IL USA.

D. G. Cahill described the use of the three omega (3*ω*) technique to measure the thermal conductivities of thin films on substrates. The 3*ω* technique involves laying down a narrow wire onto the surface of a thin-film specimen by lithographic methods. The wire, whose electrical resistance varies with temperature, acts both as a heater and as a thermometer. The name of the technique derives from the method of detection; a current at angular frequency ω is caused to flow through the wire resulting in heating at frequency 2*ω* (in addition to dc heating, which is ignored). The signal at 3*ω*, measured with a lock-in amplifier, is proportional to the ac temperature in the specimen. By proper calibration, the thermal conductivity is obtained from the frequency dependence of the signal. The technique can be used to measure the temperature dependence of the thermal conductivity. From the temperature dependence one can learn about which mechanisms are affecting the thermal conductivity. All deposited oxide films showed thermal conductivities less than bulk values. In amorphous silicon dioxide and titanium dioxide about 1 μm thick, Cahill found that atomic-scale vibrations with atomic-scale coherence lengths dominate heat transport at room temperature. The thermal conductivities of evaporated films were found to be about 66 % of the thermal conductivities of sputtered films. Thermally grown silicon dioxide had the same thermal conductivity as the bulk material. The value of the thermal conductivity could be controlled to some extent by controlling the deposition temperature and the material phase. The thermal conductivities of Si/Ge superlattices were measured as a function of period thicknesses in order to examine the effects of ITR. If ITR were important, the apparent thermal conductivity would increase with increasing period because of fewer interfaces. With periods below about 7 nm this was the case; however, above 7 nm, the thermal conductivity appeared to initially decrease and then remained approximately constant.

*Measurement of Optical and Thermophysical Thin Film Properties by Photothermal Methods*, E. Welsch and D. Ristau, Physik FSU, Jena, Germany.

E. Welsch pointed out that the two most important parameters responsible for causing laser damage in coatings are thermal conductivity and optical absorption coefficient. Photothermal methods are particular suited for measuring the thermal conductivity of optical films because they are sensitive, they permit spatial mapping, and they are noncontact in nature. The measurement of optical absorption and thermal conductivity is intertwined when employing photothermal techniques. In earlier work, an ac technique based on photothermal radiometry had been used to measure the thermal conductivity and the absorption coefficient of aluminum oxide, titanium dioxide, hafnium oxide, and tantalum pentoxide films on fused silica substrates. In more recent work, pulsed laser excitation has been used to heat the film-substrate system at the film-substrate interface. In the ideal case, the film should be transparent to the exciting radiation, while the substrate should strongly absorb it. On the other hand, the film should be opaque to the infrared radiation that is detected and the substrate should be transparent to this radiation. The pulsed technique was used to measure the thermal conductivity of films of aluminum oxide, silicon dioxide, hafnium oxide, and tantalum pentoxide deposited on germanium substrates. For a given composition, measurements were made on films of different thicknesses so that the effect of ITR could be eliminated. A comparison was made with values presented in the literature and with the bulk values. In all cases, the thin-film values were less than the bulk values. An important result pointed out by Welsch was the observation, in photothermal images, of structure that correlated with the position of laser-induced damage when the specimen was subsequently subjected to high-power irradiation.

*Thermal Transport Property Measurements of Coatings, Thin Films and Contact Conductances*, R. E. Taylor, Thermophysical Properties Research Laboratory Inc., West Lafayette, IN USA.

R. E. Taylor described the use of the laser-flash method [ASTM E1461-92] and step-heating to measure the thermal conductivity of different types of composite materials and films (or coatings). His main interest was in thermal-barrier coatings. In the laser-flash method, the front surface of a flat specimen is heated with a pulse of heat and the rear-surface temperature is monitored as a function of time. The thermal diffusivity is obtained by fitting the data to a theoretical model or by measuring the time to reach half of the maximum rear-surface temperature. In the rear-surface method, heat is applied to the front surface of the specimen at a certain instant of time and then maintained at a constant level. Here again, the diffusivity is obtained from the time dependence of the rear-surface temperature. Taylor discussed how uncertainty in certain parameters, such as film thickness, can adversely affect the uncertainty in thermal conductivity (or in diffusivity) when the laser-flash method is used. Two examples of error analyses were given when measuring the thermal conductivity of a coating on a substrate by the laser-flash method. Studies based on the laser-flash method have shown that multilayered structures behave like a homogeneous material if the ratios of the diffusivities of the constituent materials differ by factors of less than 10:1. The diffusivity of an anodized layer on an aluminum substrate was easily measured. The accuracy of the laser-flash method was validated when good agreement was found in the values obtained for thermal-barrier coatings of the same material with different thicknesses even though the temporal responses from the two specimens were vastly different. The rear-surface approach was used to measure the in-plane thermal diffusivity of CVD diamond.

*A New Method for Measuring the Thermal Conductivity of Submicron Thick Films*, S. Govorkov, W. Ruderman, M. W. Horn, R. B. Goodman, and M. Rothschild, INRAD, Northvale, NJ USA.

S. Govorkov described a new commercial instrument for measuring thin-film thermal conductivity. It is a comparator type measurement that employs photoacoustic detection. In this method, the substrate, typically 2.5 cm in diameter, is half coated with the film to be measured. The entire specimen is then overcoated with a colloidal carbon layer which acts as an absorber of the optical radiation used to induce the thermal signal. The specimen acts as one window of a photoacoustic cell. The other window is transparent to optical radiation and contains embedded within it a microphone for detecting the acoustic wave generated in the gas above the specimen when the specimen is irradiated with chopped light. A signal is measured first when the thin-film portion of the specimen is irradiated and next when the substrate portion of the specimen is irradiated. From the ratio of the two signals and known material parameters of the substrate, one can calculate the thermal resistance of the film provided that the thermal conductivity of the substrate is much greater than the thermal conductivity of the film. Thermal resistance is the ratio of the specimen thickness to thermal conductivity, therefore, the thermal conductivity can be computed if the thickness is known. The minimum measurable value of *R* is 10^−7^ m^2^ · K/W and the uncertainty of measurement at *R* = 10^−5^ m^2^ · K/W is claimed to be 5 %. Sample thermal conductivity data taken with silicon dioxide and silicon nitride films deposited on silicon wafers agree with prior reported measurements.

*Thin Film Thermophysical Property Characterization by Scanning Laser Microscope*, G. Chen, T. Borca-Tasciuc, and M. Neagu, Duke University, Durham, NC USA.

T. Borca-Tascius described a method for obtaining high-resolution images of thermal waves in a thin film deposited on a substrate. The data can be used to calculate the diffusivity of the film with a possibility for measurement of the anisotropy of thermal diffusivity. As described, the method requires a metallic film deposited on a transparent substrate. A fine wire held in contact with the top surface of the film acts as one arm of a thermocouple and the film is the other arm. AC heating occurs when a chopped laser beam is focused through the substrate onto the film-substrate interface. Micrometer scale resolution was demonstrated on a gold film deposited on a glass substrate by scanning the position of the focal spot relative to the position of the thermocouple. However, a reliable value of the thermal diffusivity had not yet been achieved.

*Thin Film Discussion Session*, Chairman, G. White, NIST.

The discussion session dealt with several issues. One important issue was: how do we know that the numbers we are quoting are really any good? No specific answer was found but participants seemed to agree that this question might be resolved by holding a round-robin. Many participants appeared to be willing to participate in a round-robin or interlaboratory comparison using their own measurement procedure. Silicon dioxide prepared by oxidation of silicon was considered to be the best low-conductivity material for both a round-robin and as a reference material. No consensus was found for a high thermal conductivity material. There was a clear disagreement on how the thermal conductivity of thin films depends on film thickness. Another issue that arose was the meaning of an interface. Some thought that the definition should apply only to sharp boundaries, while others were of the opinion that an interface could actually be a layer of small, yet finite, thickness that exists between a film and a substrate. Clearly, the nature of such an interface is important to thermal transport and authors should define what they mean by an interface. The application areas of interest to the Workshop attenders appeared to be microelectronics, thermal-barrier coatings, and high-power laser optics; thus, the need for measurements of thin-film thermal conductivity concentrated on these areas. There was general agreement about the need for better measurement reliability and reproducibility.

## 3. Thermal Conductivity Measurements on CVD Diamond

The thermal conductivity of diamond is receiving considerable attention because the material possesses a higher thermal conductivity than any other material at room temperature. Nearly all of the talks dealt exclusively with measurement techniques and measurements except for the talk by H. Verhoeven.

*Measurements of Thermal Conductivity and Diffusivity in CVD Diamond*, J. E. Graebner, Lucent Technologies, Murray Hill, NJ USA.

In a session that was not part of the Workshop, J. Graebner of Lucent Technologies gave an invited presentation that summarized recent thermal conductivity measurements he had made on thin plates of CVD diamond. He has used the dc heated-bar technique to measure the in-plane or parallel component of the thermal conductivity and the laser-flash technique to measure the normal component of the thermal conductivity. The principal conclusions are: CVD diamond can be made with a thermal conductivity equal to that of pure single-crystal diamond; the thermal conductivity of as-grown diamond plates is highly inhomogeneous, with the thermal conductivity at the nucleation surface being significantly smaller than the thermal conductivity at the growth surface; the thermal conductivity is anisotropic with the parallel and normal components depending differently on position relative to the growth surface. These effects have been attributed to the conical columnar morphology of diamond grains in the plates.

*A Modification of Ångström’s Method That Employs Photothermal Radiometry to Measure Thermal Diffusivity: Application to CVD Diamond*, N. M. Balzaretti^2^ and A. Feldman, National Institute of Standards and Technology.

N. M. Balzaretti described a modification of Ångström’s method for measuring the in-plane thermal diffusivity of long, thin bars of CVD diamond. Ångström’s method is based on one dimensional ac heat flow along a long bar specimen. The bar typically is heated with an ac heat source at one end; the magnitude of the temperature, |*T*(*x*)|, and the phase, *φ*(*x*), of the thermal wave that propagates down the bar are measured at several points along the bar. The measurement procedure described by Dr. Balzaretti consists of heating a specimen near its center with an argon-ion laser beam and measuring the infrared radiation emitted by the specimen as a function of distance from the heated region. The one-dimensional theory is adequate to analyze the experimental data. Further simplification occurs if the specimen is sufficiently long, for then ln |*T*(*x*)| and *φ*(*x*) will depend linearly on *x*; the diffusivity can be obtained either from d(ln |*T*(*x*)|)/d*x* or from d*φ*(*x*)/d*x* provided that the region used for computing either slope is sufficiently far from the heating source and from the specimen ends. No significant differences were found in diffusivities calculated from the phase data and diffusivities calculated from the magnitude data. Thus, losses to the surroundings, such as radiative loss, which affect phase and magnitude differently, were considered to be negligible. The values of diffusivity obtained agree reasonably well with the mean values calculated from measurements made by several other laboratories that had used a different modification of Ångström’s method on the very same specimens as part of an interlaboratory comparison.

*Modified AC Calorimetry Using a Modulated Laser Beam Energy Source*, R. Kato, A. Maesono, R. P. Tye, and I. Hatta, Sinku Riko, Inc. Yokohoma, Japan and Nagoya University, Nagoya, Japan.

R. P. Tye described a new commercial ac calorimeter. This instrument, derived from an earlier instrument developed by Hatta, employs a modified Ångström’s method. The instrument operates best with a bar shaped specimen. The heat source is a 30 mW laser diode operating at 680 nm; the beam is scanned perpendicular to the long axis of the specimen with a multi-facet mirror with a sweep frequency of 320 Hz. The sweep frequency greatly exceeds the measurement frequency, which can be between 0.01 Hz and 2 Hz. The measurement frequency is chosen so that the thermal diffusion length is between 2 mm and 4 mm. The detector is a thermocouple cemented to the back (unilluminated side) of the specimen. Two sets of specimens were measured: the first consisted of specimens made of 99.99 % oxygen free copper, 99.99 % Ni, 304 stainless steel, a ceramic, glassy carbon GC-20, Pyrex glass and Plexiglas; the other consisted of the three CVD diamond specimens discussed in the previous paragraph. From the first set of specimens, the values reported for the three metals, the ceramic, and the glassy carbon agree with published data within 5 %. However, the data on the lowest diffusivity materials, Pyrex and the Plexiglas, were not given because the values obtained were not considered acceptable. The problem was believed to be due to the influence of air adjacent to the specimens. Measurements in vacuum are planned. Data from the CVD diamond specimens were found to be in good agreement with measurements made on the earlier instrument and in reasonably good agreement with the measurements discussed in the previous paragraph.

*Influence of the Microstructure on the Thermal Properties of Polycrystalline Diamond Films of Micrometer Thickness*, H. Verhoeven, A. Flöter, H. Reiβ, R. Zachai, Daimler-Benz AG, New Materials, Postfach 2360, 89013 Ulm, Germany and D. Wittorf, J. Jäger, Institute für Festkörperforschung, Forschungszentrum Jülich, 52425 Jülich, Germany.

H. Verhoeven discussed the effect of microstructure on the ITR, the thermal conductivity, and the thermal conductivity anisotropy of CVD diamond films 0.5 μm to 4.0 μm thick. Different morphologies were produced by the preparation of films under different deposition conditions in a microwave plasma deposition system. Two categories of films were defined, (1) films grown at high temperature (800 °C) falling into two subcategories: random nanometer grained and highly oriented, and (2) films grown at low temperature (500 °C and 550 °C) called columnar grained. Micro-Raman spectroscopy indicated that the quality of the columnar grained films did not depend on film thickness, whereas the quality of the highly oriented films improved significantly with thickness. A highly oriented film grown at high temperature showed a SiC interlayer 10 nm thick between the film and the substrate, whereas a film grown at the low temperature showed an amorphous interlayer 3 nm thick. Two techniques were used to measure thermal conductivity and ITR: photothermal displacement at transient thermal gratings and thermoreflectance. The ITR of the highly oriented films was essentially independent of layer thickness, suggesting that the ITR dominated the heat transport. The other categories of films showed an ITR that increased with film thickness indicating a more uniform phase purity, a result which was consistent with the Raman measurements. An ITR of about 2×10^−9^ m^2^ · K/W to 4×10^−9^ m^2^ · K/W was measured in all cases, which was attributed to near-interfacial disorder. This is an order of magnitude greater than would be expected for a perfect solid-solid interface. In addition, it was found that the normal-to-plane thermal conductivity was 9 to 18 times the in-plane thermal conductivity in the columnar films and in the highly oriented films. The randomly oriented films showed a much smaller anisotropy. The large anisotropy is attributed to the columnar structure; phonons responsible for heat transport will encounter many fewer defects along the columns than across the columns.

*Thermal Conductivity Measurements on CVD Diamond Using Graphitic Microheaters Prepared by Laser Scribing*, E. Wörner, C. Wild, W. Müller Sebert, and P. Koidl, Fraunhofer Institut für Angewandte Festkörperphysik, Freiburg, Germany.

E. Wörner described thermal conductivity measurements on CVD wafers using graphite microheaters prepared by laser scribing. Laser beams typically used to cut diamond plates have been used to graphitize thin strips on the surface of CVD diamond wafers. Graphitization usually occurs automatically when cutting the wafers so that frequently no special preparation procedure may be necessary to produce a heater. Heaters of this type were used as sources for a modified laser-flash technique to determine the thermal conductivity of diamond wafers. A series of thermal conductivity measurements were made on several specimens cut from a single large diamond wafer. On a rectangular shaped wafer, a line source scribed along one edge was heated with an electrical pulse and infrared radiation was collected from the opposite edge and focused onto a HgCdTe detector. On a circular wafer, a circular heater scribed along the outer perimeter was heated with an electrical pulse and infrared radiation was collected from the center of the wafer and focused onto the detector. For comparison, measurements on a rectangular specimen were also made by a dc heated bar technique that is quite accurate. The values obtained by all of the techniques agreed within 10 %.

*Laser Flash Diffusivity Measurement of Diamonds: “Some Experimental Specificities,”* B. Remy, D. Maillet, and S. Andre, Laboratoire d’Énergetique et de Mécanique Théorique et Aplliquée - INPL-UHP Nancy I — URA CNRS 875 -02, avenue de la forêt de Haye, BP 160, 54 504 Vandoeuvre-Lès-Nancy Cedex, France.

B. Remy discussed the use of the laser-flash method for measuring the thermal diffusivity of unsupported nondiamond (material not specified) and CVD diamond coatings. The coatings were several tenths of millimeters thick. A pulsed beam from a Nd-glass laser illuminated the front surface of the specimen and the infrared signal emitted by the rear surface of the specimen was measured by a cooled HgCdTe photovoltaic detector. Several experimental requirements were discussed including the need to coat transparent specimens in order to prevent unwanted radiation from reaching the detector. The response time of the detection system including detector and amplifiers had to be sufficiently short because the signal rise time to half maximum could be shorter than 5×10^−5^ s. A particular component described by Remy was a copper tube that collected infrared radiation from the emitting surface of the specimen which is then focused onto the detector with a germanium lens. Use of the one-dimensional theoretical model simplifies the data analysis procedure enormously; if the laser beam is not uniform, a two-dimensional model might be required. The copper tube appeared to partially correct for higher-dimensional effects caused by nonuniform illumination of the specimen. Experimental data could be fit quite well to the theoretical model yielding reasonable values of thermal conductivity. However, there was no basis for determining the accuracy of the results.

*Discussion of Diamond Round-Robin II*, Chairman, J. Graebner, Lucent Technologies.

The final session was devoted to a discussion of a round-robin comparison of thermal conductivity measurements on a set of CVD diamond specimens and several other specimens of lower thermal conductivity. This was a second round-robin which had been organized because in a prior round-robin, large interlaboratory differences had been obtained which had been attributed to specimen inhomogeneity. In this second round-robin, CVD diamond specimens 40 mm long by 7 mm wide by 0.2 mm to 0.7 mm thick had been prepared for measurement. In order to insure better homogeneity, about 150 μm was ground off the nucleation surface of the wafers from which the specimens were fabricated. Several other specimens having the same nominal dimensions were prepared from ceramic materials having lower thermal conductivity values. After several laboratories performed measurements on these long specimens, the specimens were cut in half making the long dimension 20 mm and doubling the number of specimens. Measurements were then done by the other participants. John Graebner summarized the results to date. Unfortunately, large interlaboratory differences in thermal conductivity (or diffusivity) values were still reported. There was good consistency among the dc measurements, however, the ac measurements showed much greater variability. Further work needs to be done to ascertain the sources of the discrepancies.

## Footnotes

1 Permanent address: Instituto de Fisica - UFRGS - Porto Alegre, RS - 91501-970 - Brazil

## 4. Appendix

Questions and Responses to Voluntary Questionnaire.

1. What industry do you associate yourself with and what is its relationship to your current position? (Example: University researcher performing research related to the semiconductor industry.)
  **Table:**
  5	govt. researchers		measurements and/or standards development
  1	“	“	high-power semiconductor laser industry
  5	university researchers		semiconductor industry
  2	“	“	emeritus
  1	“	“	student
  1	“	“	optics and semiconductors
  1	“	“	high temperature applications
  1	“	“	electronics and coatings
  2	“	“	high-power laser optics
  1	“	“	technical information provider
  1	president of a testing facility		
  1	manager of research at an aerospace company, thermal barrier coatings		
  1	consultant to thermal equipment manufacturer		
  1	researcher at automobile manufacturer, high-power electronics		
  1	researcher at a research center		
2. Do you produce or do you use thin-film thermal conductivity data and how does this relate to your position? (Example: produce thermal conductivity data for research, use thermal conductivity data for design of electronic circuits).
  **Table:**
  11 produce data for research and/or standards
  5 use data for research, data compilations, modeling
  7 produce and use data for research and design
  1 establishes measurement technology for thin films
3. Rate the relevance of thin-film thermal conductivity measurement to your activities on a scale of zero (irrelevant) to 5 (essential).
  **Table:**
  Score	Number of replies
  1	1
  2	0
  3	0
  3–4	1
  4	9
  4–5	1
  5	12
4. Would you be willing to collaborate with others in the area of thin-film thermal conductivity measurement? (Examples: round-robin interlaboratory measurement comparisons, standards development, organizing future Workshops)
  **Table:**
  Yes	20
  Maybe	2
  No	1
  No reply	2
5. Rate the usefulness of this Workshop on a scale of 0 (waste of time) to 5 (extremely useful).
  **Table:**
  Score	Number of replies
  1	0
  2	0
  3	0
  3–4	0
  4	7
  4–5	2
  5	16
6. Do you see a need for additional Workshops in the future? If so, how often should they convene?
  **Table:**
  Frequency (years)	Number of yes of replies
  1	5
  1–2	4
  2	6
  2–3	2
  3	3
  3–4	0
  4	0
  4–5	0
  5	1

